# Effects of high tibial osteotomy combined with arthroscopy on local inflammation degree and gait activity index in patients with medial knee osteoarthritis

**DOI:** 10.12669/pjms.39.1.6846

**Published:** 2023

**Authors:** Qinghan Li, Haitao Wang, Dalin Wang

**Affiliations:** 1 Qinghan Li, School of Clinical Medicine, Beihua University, Jilin, Jilin Province, 132000 P.R. China; 2 Haitao Wang, School of Clinical Medicine, Beihua University, Jilin, Jilin Province, 132000 P.R. China; 3 Dalin Wang, School of Clinical Medicine, Beihua University, Jilin, Jilin Province, 132000 P.R. China

**Keywords:** Medial knee osteoarthritis, Arthroscopy, High tibial osteotomy, Degree of inflammation, Gait activity index

## Abstract

**Objective:**

To analyze the effects of high tibial osteotomy (HTO) combined with arthroscopy on the degree of inflammation and the gait activity indexes of patients with medial knee osteoarthritis.

**Methods:**

We collected the records of 112 patients with medial knee osteoarthritis treated in the Department of Orthopedics of our hospital from June 2019 to June 2021. We divided the data into two groups: the control group included those of 54 patients who had received simple HTO and the observation group included those of 58 patients who had undergone HTO combined with arthroscopy. We assessed clinical efficacies, degrees of inflammation and gait activity indexes of the two groups to compare treatment outcomes.

**Results:**

The percentage of excellent and good outcomes in the observation group (89.66%) was significantly higher than that in the control group (66.67%; *p* < 0.05). One year after the operation, the serum and synovial fluid levels of interleukin-1β (IL-1β), interleukin-6 (IL-6) and interleukin-17 (IL-17) in the observation group were significantly lower than those in the control group (*p* < 0.05). Moreover, the double support phase was significantly lower in the observation group than in the control group, while the step length, speed and frequency were significantly higher in the observation group than in the control group (*p* < 0.05).

**Conclusions:**

HTO combined with Arthroscopy in patients with medial knee osteoarthritis improves the curative effect and the degree of inflammation, and it promotes the recovery of gait activity indices.

## INTRODUCTION

Medial knee osteoarthritis is a common chronic joint degenerative disease caused by articular cartilage changes and secondary hyperosteogeny. The disease occurs mainly in middle-aged and elderly individuals affecting the affected joint function and resulting in an abnormal gait that is difficult to treat.^1^,^2^ Surgery is an effective treatment for medial knee osteoarthritis, and it commonly includes a HTO. This procedure can effectively correct the normal force line of the lower limbs, improve the femoral tibial angle, alleviate the medial osteoarthritis pressure, and maintain its balance.^3^

Thus, HTO can effectively alleviate the patient’s condition; however, effectively dealing with the internal lesions of the knee joint is difficult, and the improvements are limited after this procedure on its own.^4^ Arthroscopy can repair damaged meniscii and fully remove the abnormal synovial hyperplasia, removing these pain causing elements from the joint cavity and promoting knee function improvement and promote joint function recovery.^5^ Some studies have been conducted on HTO combined with arthroscopy for the treatment of patients with medial knee osteoarthritis, but reports on inflammation improvement and gait activity indicators are scarce.

Our objective was to analyze the effects of HTO combined with arthroscopy on the degree of inflammation and the gait activity indexes of patients with medial knee osteoarthritis.

## METHODS

We extracted data from the records of 112 patients with medial knee osteoarthritis treated with the same technique by the same surgical team from June 2019 to June 2021 in the Department of Orthopedics of our hospital.

### Inclusion criteria:

- Lesion confirmed by X-ray examination, and signs and symptoms consistent with the diagnostic criteria.^6^
- Pain in the inner side of the knee joint that did not go away after six months of conservative treatment
- Varus deformity of the affected knee joint evidenced by imaging studies
- Age ≥ 18 years
- Single knee disease, Varus deformity of knee joint < 15°, range of motion ≥110°, and flexion contracture < 5°
- The Kellgren Lawrence classification ranged between II and III, and the international cartilage repair classification system (ICRs) was ≤ III.
- Patients with complete follow-up data one year after operation

### Exclusion criteria:

- Other arthritis types, such as infectious arthritis or rheumatoid arthritis;
- Incomplete follow-up data
- Mental or coagulation dysfunction
- Malignant tumor combined with patellar and lateral compartment arthritis.

### Ethics Approval:

The study was approved by the medical ethics committee of School of Clinical Medicine, Beihua University (Approval no. BH-LL-20220105, Date: 2021 Jan 13).

### HTO combined with arthroscopy:

The patients were administered general anesthesia and a tourniquet was put in place. Arthroscopy was initiated to explore the affected knee joint through standard anterior external and anterior internal approaches.

Any proliferative synovial tissue in the joint cavity was cleaned and free bodies were removed. Patients with meniscus injuries not exceeding the red and white area, or with worn edges of the meniscus underwent meniscaloplasty.

In addition, patients with tibial plateau cartilage damage and medial femoral damage with an ICR grade between I and II underwent chondroplasty. Patients with ICR Grade-III underwent bone marrow stimulation with micro fracture tools. After the arthroscopic exploration, the surgeon performed a HTO, that is, the medial edge of the tibia was located, and an arc incision was made at the upper anterior and lower parts of the tibia to fully expose patellar tendon insertion. Medial opening wedge osteotomy for all patients were performed by the same surgical team and the surgical techniques as been detailed in previous studies^7^, ^8^

**Fig-1:**
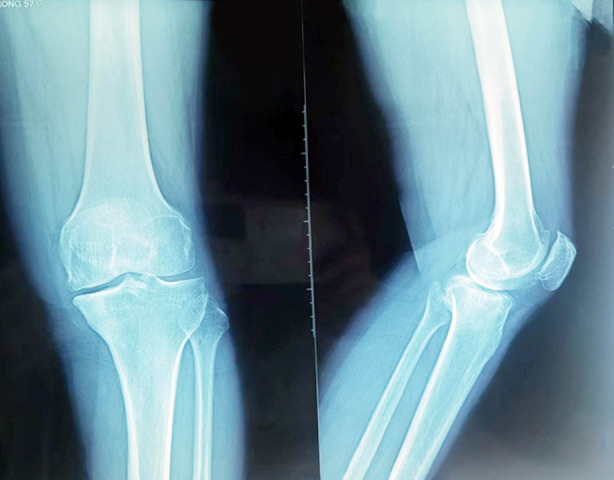
Preoperative radiographs of medial knee osteoarthritis.

**Fig-2:**
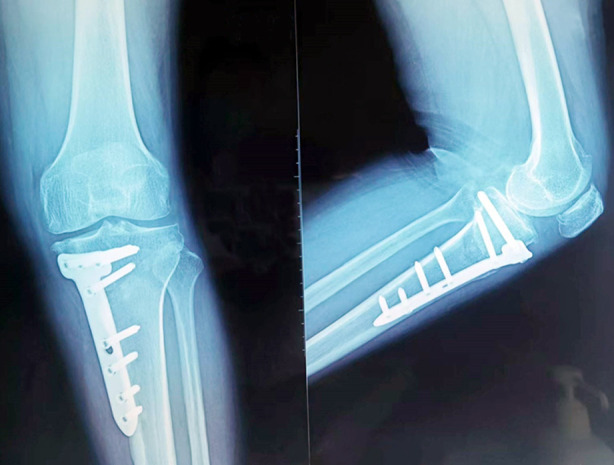
Radiographs of patients with medial knee osteoarthritis after HTO combined with arthroscopy.

Having completed the procedure, the surgeon fully flushed the operation area, routinely retained the drainage tube, closed the incision layer by layer and compressed with sterile dressings and bandages. After the operation, the patients were given antibiotic treatment, infection prophylaxis and rehabilitation training. Fig.1 and Fig.2.

### Basic patient information and treatment related indicators:

One year after operation, the osteoarthritis improvement was assessed by calculating the Western Ontario and McMaster Universities Arthritis Index (WOMAC) score and classified as excellent (WOMAC score improvement rate ≥75%), good (improvement rate between 50% and 75%), moderate (improvement rate between 25% and 50%), or poor (improvement rate <25%).^9^

The levels of interleukin-1β (IL-1β), interleukin-6 (IL-6) and interleukin-17 (IL-17) before and one year after operation were measured from 5-ml fasting elbow vein blood and a 3-ml joint synovial fluid samples by enzyme-linked immunosorbent assays (Boyao Biotechnology kit used as per manufacturer’s instructions). 3) We collected the data on biphasic support, step length, gait speed and gait frequency one year after the operation.

### Statistical analysis:

We used SPSS22.0 for data processing, we represented non-grade count data as counts and percentages [n (%)] and used the *χ^2^* method for inspection. We represented measurement data as means and standard deviations (*χ̄*±*S*). T-tests were performed. We considered all (*p* < 0.05) as indicative of statistically significant differences.

## RESULTS

We found similar general data between the two groups (*p* > 0.05; Table-I). The combined rates of excellent and good osteoarthritis improvement in the observation group was 89.66%, a rate higher than the 66.67% of the control group (*p* < 0.05; Table-II). Before the operations, the levels of IL-1β, IL-6 and IL-17 in serum and synovial fluid between the two groups were similar (*p* > 0.05). However, one year after the operation, the levels of IL-1, IL-6 and IL-17 in serum and joint synovial fluid of the two groups were lower than before, with the levels in the observation group being significantly lower than those in the control group (*p* < 0.05). Table-III.

**Table-I:** Comparison of general information between the two groups.

Group	n	Gender (Male/Female)	Age (Year)	Affected side (n)		Kellgren-Lawrence classification (n)	
	
				Left knee	Right knee	Grade II	Grade III
Control group	54	31/23	51.81±12.17	26	28	35	19
Observation group	58	27/31	52.96±11.78	33	25	31	27
x^2^/t	-	1.320	0.445	0.859		1.493	
p	-	0.251	0.658	0.354		0.222	

**Table-II:** Comparison of clinical efficacy scores between the two groups [*n* (%)].

Group	n	Excellent	Good	Middle	Poor	Excellent rate
Control group	54	15 (27.78)	21 (38.89)	15 (27.78)	3 (5.56)	36 (66.67)
Observation group	58	23 (39.66)	29 (50.00)	5 (8.62)	1 (1.72)	52 (89.66)
χ^2^/t	-	-	-	-	-	8.833
p	-	-	-	-	-	0.032

**Table-III:** Comparison of the pain stress levels between the two groups (*χ̅*±*s*,pg/ml)

Index	Group	n	IL-1β			IL-6	IL-17	

			Before operation	One year after operation	Before operation	One year after operation	Before operation	One year after operation
Serum	Control group	54	4.83±0.99	4.06±1.05^a^	3.98±1.21	3.27±1.17^a^	5.64±1.26	4.42±1.15^a^
	Observation group	58	4.98±1.00	3.29±0.89	3.89±1.24	2.37±1.05^a^	5.56±1.40	3.70±1.28^a^
t		-	0.765	4.229	0.366	4.240	0.347	3.115
p		-	0.446	<0.001	0.715	<0.001	0.729	0.002
Synovial fluid	Control group	54	6.01±1.39	4.86±1.18^a^	5.08±1.48	3.97±1.23^a^	7.09±1.72	6.10±1.51^a^
	Observation group	58	5.93±1.55	4.14±1.33^a^	4.93±1.56	3.07±1.31^a^	6.93±1.70	4.91±1.38^a^
t		-	0.273	3.008	0.532	3.696	0.482	2.915
p		-	0.785	0.003	0.596	<0.001	0.631	<0.001

***Note***:

a compared with this group before surgery *p* < 0.05.

In addition, we had found similar indices of preoperative biphasic support phase, step length, gait speed and gait frequency between the two groups (*p* > 0.05). However, one year after operation, although the gait activity indices of the two groups had improved compared with those before the operation, the double support phase of the observation group was significantly lower and the step length, pace and frequency significantly higher than those in the control group (*p* < 0.05). Table-IV

**Table-IV:** Comparison of tibial plateau deformity and joint range of motion scores between the two groups (*χ̅*±*s*)

Group	n	Dual support phase (%)		Step size (cm)		Pace (cm/s)		Cadence (Steps/min)	
			
		Before operation	One year after operation	Before operation	One year after operation	Before operation	One year after operation	Before operation	One year after operation
Control group	54	34.18±2.80	30.18±2.47^a^	33.31±4.36	52.94±5.29^a^	83.13±5.64	90.20±6.09^a^	82.18±5.83	91.26±6.42^a^
Observation group	58	34.27±3.04	26.22±2.79^a^	33.53±3.83	62.81±4.88^a^	83.72±6.28	99.00±7.02^a^	83.69±6.04	105.55±6.53^a^
t	-	0.164	7.916	0.284	10.268	0.525	7.056	1.339	11.666
p	-	0.870	<0.001	0.777	<0.001	0.600	<0.001	0.183	<0.001

***Note***:

a compared with this group before surgery *p* < 0.05.

## DISCUSSION

Knee osteoarthritis is a common bone and joint disease characterized by articular cartilage degeneration or damage with an increasing incidence in recent years^10^, ^11^. At present, stepped-care approach is widely employed in the treatment of patients with for, that is, surgical treatment would be performed for those who do not respond well to conservative treatment such as physical therapy and medications.

The surgical treatments for medial knee osteoarthritis include unicompartmental knee joint replacement, HTO and arthroscopic surgery.^12^ Among the surgical options, HTO is a knee preserving approach, which has a high knee joint survival rate and is a relatively simple operation. By correcting the lower limb force line, the patient’s knee joint function can be effectively preserved.^13^ However, Ji W et al^14^ found that the capability of HTO to correct the intra-articular varus deformity is limited when only a HTO is used to treat patients with medial knee osteoarthritis. Meanwhile, arthroscopic surgery can clean and repair the damaged tissues in the patient’s joint cavity, with minimal trauma and quick recovery.^15^ Therefore, combining the procedure with an arthroscopic surgery is recommended.

Zhou X et al^7^ treated patients with medial knee osteoarthritis with arthroscopy and HTO. Their results showed that the knee function of the patients was improved significantly, the degree of pain was relieved, and the operation also promoted the regeneration of the articular cartilage. In our study, we found that the percentage of patients with excellent and good osteoarthritis improvement rates were higher in the observation group in the control group (p <0.05), indicating that this treatment modality can improve the outcomes for these patients, a finding consistent with the research results of Han CX et al.^16^

Arthroscopy and HTO have complementary advantages. During arthroscopic operations, surgeons remove mechanical irritants such as exfoliated cartilage, proliferative synovium, loose bodies and torn meniscusii caused by joint degenerative lesions, and they repair the damaged cartilage and suture torn meniscusii. These steps promote the effective recovery of the internal environment of the joint cavity, alleviate the pain, improve the functional limitation degree, and create favorable conditions for the repair of degenerative cartilage tissue.

An additional HTO can move the weight-bearing mechanical axis of the lower limb outward, correct the Varus deformity of the knee, alleviate the stress level of the medial compartment of the knee, promote the timely recovery of cartilage tissues, maintain a normal internal pressure, reduce the medial bone pressure, promote the improvement of the local microcirculation function, and prevent the accelerated degeneration of the joint, thereby improving the curative effect for patients.^17^

The local inflammatory response of patients with medial knee osteoarthritis is increased, and inflammatory factors such as IL-1β, IL-6 and IL-17 are directly involved in the occurrence and progression of the disease. Elbaz A et al found that the gait of patients with knee osteoarthritis was significantly abnormal.^18^ The double support phase, step length, pace and step frequency are important indicators reflecting human gait activity.

In our study, we found that a year after the operation, the levels of IL-1β, IL-6 and IL-17 in serum and synovial fluid of the patients in the observation group were lower than those of the patients in the control group, and that the double support phase was also lower than that in the control group, while the step length, pace and frequency were higher than those in the control group (*p* <0.05), indicating that HTO combined with arthroscopy can also diminish the inflammation and promote the recovery of gait activity indicators. Our results are similar to those of Zhao B et al.^8^

For patients with medial knee osteoarthritis undergoing HTO combined with arthroscopy, anti-inflammatory plus antibiotic treatment is provided to inhibit inflammatory mediators in the joint cavity after operation. Moreover, the healing gets promoted by effectively correcting the joint deformity and using the cancellous bone area in the osteotomy site and the delayed healing of bone can be prevented during the operation. In addition, the damaged tissue in the joint can be removed during the operation, the weight-bearing can be shifted laterally, and thus repair the soft tissue and prevent the appearance of inflammatory mediators.

In all, the reduction in the expression of inflammatory factors in serum and joint synovial fluid, the preservation of the proprioception of the knee joint, the maintenance of the balance of soft tissue and lower limb force line, and the promotion of early rehabilitation training by patients after the operation are the factors that come together to promote the rehabilitation of the limb function and improve the gait activity index.

### Limitations:

This was a single center retrospective study with a small number of cases, few observation indicators, and no follow-up investigation on the long-term efficacy of the treatment. Prospective multi center and large sample studies are needed to validate our results.

## CONCLUSION

The effect of HTO combined with arthroscopy in patients with medial knee osteoarthritis is beneficial, it can improve the curative effect, diminish the local inflammation, and promote the recovery of gait activity indices when compared to the results of HTO alone.

### Authors’ Contributions:

**QL** conceived and designed the study.

**HW and DW** collected the data and performed the analysis.

**QL** was involved in the writing of the manuscript and is responsible for the integrity of the study.

All authors have read and approved the final manuscript.
